# Experimental and Numerical Study of Non-Explosive Simulated Blast Loading on Reinforced Concrete Slabs

**DOI:** 10.3390/ma16124410

**Published:** 2023-06-15

**Authors:** Zhixiang Xiong, Wei Wang, Guocai Yu, Jian Ma, Weiming Zhang, Linzhi Wu

**Affiliations:** 1 Key Laboratory of Advanced Ship Materials and Mechanics, Harbin Engineering University, Harbin 150001, China; xiongzx@hrbeu.edu.cn (Z.X.);; 2 Department of Engineering Mechanics, College of Aerospace and Civil Engineering, Harbin Engineering University, Harbin 150001, China; 3 Key Laboratory of Impact and Safety Engineering, Ningbo University, Ministry of Education, Ningbo 315211, China; 4 Center for Composite Materials, Harbin Institute of Technology, Harbin 150080, China

**Keywords:** blast simulator, impact loading, reinforced concrete slabs, parameter studies

## Abstract

This study presents a non-explosive method for simulating blast loading on reinforced concrete (RC) slabs. The method involves using a newly developed blast simulator to apply a speedy impact load on the slab, which generates a pressure wave similar to that of an actual blast. Both experimental and numerical simulations were carried out to evaluate the effectiveness of the method. The experimental results showed that the non-explosive method can produce a pressure wave with a peak pressure and duration analogous to those of an actual blast. The numerical simulations also showed good agreement with the experimental results. Additionally, parameter studies were conducted to evaluate the effects of the rubber shape, the impact velocity, the bottom thickness, and the upper thickness on the impact loading. The results indicate that pyramidal rubber is more suitable as an impact cushion for simulating blast loading than planar rubber. The impact velocity has the widest range of regulation for peak pressure and impulse. As the velocity increases from 12.76 to 23.41 m/s, the corresponding range of values for peak pressure is 6.457 to 17.108 MPa, and for impulse, it is 8.573 to 14.151 MPa∙ms. The variation in the upper thickness of the pyramidal rubber has a more positive effect on the impact load than the bottom thickness. With the upper thickness increasing from 30 mm to 130 mm, the peak pressure decreased by 59.01%, and the impulse increased by 16.64%. Meanwhile, when the bottom part’s thickness increased from 30 mm to 130 mm, the peak pressure decreased by 44.59%, and the impulse increased by 11.01%. The proposed method provides a safe and cost-effective alternative to traditional explosive methods for simulating blast loading on RC slabs.

## 1. Introduction

Critical infrastructure in sectors, such as energy, communications and government, are highly vulnerable to the threat of improvised explosives, as demonstrated by the ongoing terrorist attacks around the world. To ensure the security and stability of societies, governments are faced with a serious challenge and there is an urgent need for a cost-effective testing tool to support the study of blast effects in the near zone of structures, so that new blast-resistant structures can be developed, validated and deployed more quickly.

Blast testing has long been the primary means of studying close-in blast loading [1,2,3]. The explosive is capable of exerting a pressure of several tens to hundreds of megapascals on the specimen within a short period of time after detonation, and such test conditions are difficult to replicate by other means. Schenker [4] conducted full-scale blast tests on concrete slabs to obtain dynamic response test data for concrete elements and verified these via numerical calculations. Dharmasena et al. [5] used stainless steel honeycomb sandwich panels and investigated the extent of damage to specimens at 100 mm blast distance and trinitrotoluene (TNT) doses of 1.0, 2.0 and 3.0 kg respectively. However, this research tool is characterized by the difficulty of measuring dynamic mechanical parameters, large scatter, low repeatability and poor visibility of component damage. Therefore, non-explosive methods that are safe, controllable and can be studied in the laboratory, such as the gas gun, the shock tube and blast simulator, has emerged. Since the blast shock wave causes damage to the human brain at pressures lasting a few milliseconds in the approximate range of 68.95 to 690.48 Pa [6], the gas gun is well suited for shock wave testing in this low-pressure range. Bartyczak [7] improved the existing gas gun method and investigated the impact resistance of helmet materials. Schleyer [8] summarized a series of tests with shock tubes on large structures, which proved that the tubes were suitable for simulating the loads generated by far ranges explosions. In order to investigate the effect of concrete strength on the dynamic response of concrete slabs under blast loading, Thiagarajan [9] conducted impact tests on four types of concrete slabs using a shock tube to simulate air blast loading.

To solve the problem of not being able to load full-scale components with gas guns, and to compensate the limitations of shock tubes for near-zero blast studies, the first blast simulation device [10] has been developed (2006) at the University of California, San Diego (UCSD) for the investigation of the blast resistance of different walls, columns and composite structures. Oesterle [11] investigated the impact resistance of concrete masonry walls containing different reinforcements at impulse of 1000–20,000 Pa·s. Gram [12] carried out impact and blast tests on reinforced concrete columns and compared the results of the two tests, which showed good similarity in deformation and failure modes of the specimens. Rodrigueznikl [10] first captured the formation of shear cracks and the spalling of the concrete protective layer when studying reinforced concrete columns under 6800–15,700 Pa·s impulse. This impulse corresponds to the blast load of 560 kg of high explosive on a vehicle at 0.9 m from the ground and 3.5–6.1 m blast distance. Freidenberg [13] carried out impact tests on high-strength prototype walls, all of which simulated blast distances of several meters and charge sizes of several hundred kilograms equivalents, and produced planar shock waves similar to those produced by car bombs. Huson [14] demonstrated the use of water bags to apply loads to composite sandwich members and member joints. Wolfson [15] briefly discuss the dynamic response and damage patterns of honeycomb structures under the action of a proximity blast by firing different types of impact modules. The European Commission Joint Research Centre (JRC) has proposed the development of explosion simulation capabilities similar to those of the United States. The European Laboratory for Structural Assessment (ELSA) has constructed a new testing facility called the electronic blast simulator (e-BLAST) [16,17,18], which uses servo-controlled drive technology to simulate the loading of structures by air shock waves without the use of explosives. The facility uses three synchronized electric linear motors to accelerate the impact module and load the structure under study. Elastic materials are placed between the impact mass and the structure to maintain consistency with relevant air shock wave pressures. This method will not only describe the effects of explosions on structural systems but also evaluate reinforcement and retrofitting techniques for buildings and bridges to resist terrorist bomb attacks. It will also help investigate the problem of progressive collapse, where local damage spreads disproportionately and leads to overall destruction, as seen in the Oklahoma City bombing.

Currently, there is a lack of research on RC slabs using blast simulators, and the range of impact intensities for simulated blast loading is not sufficient to cover a wide range of blast environments. To address this issue, this paper conducted nine impact tests on reinforced concrete slabs using the newly developed VMLH Blast Simulator. The study also included numerical calculations to investigate the effects of rubber shape, impact velocity, and the bottom and top thicknesses of the pyramidal rubber on the impact loading. Additionally, two equivalence criteria are proposed and used to compare the test results with ideal blast loading. The VMLH Blast Simulator has the ability to safely and precisely apply blast-like loading to components within a controlled laboratory setting, addressing the limitations of traditional explosion testing in terms of data collection, testing duration, and expenses.

## 2. Experiment Program

### 2.1. Non-Explosive Blast Simulator

**Figure 1:**
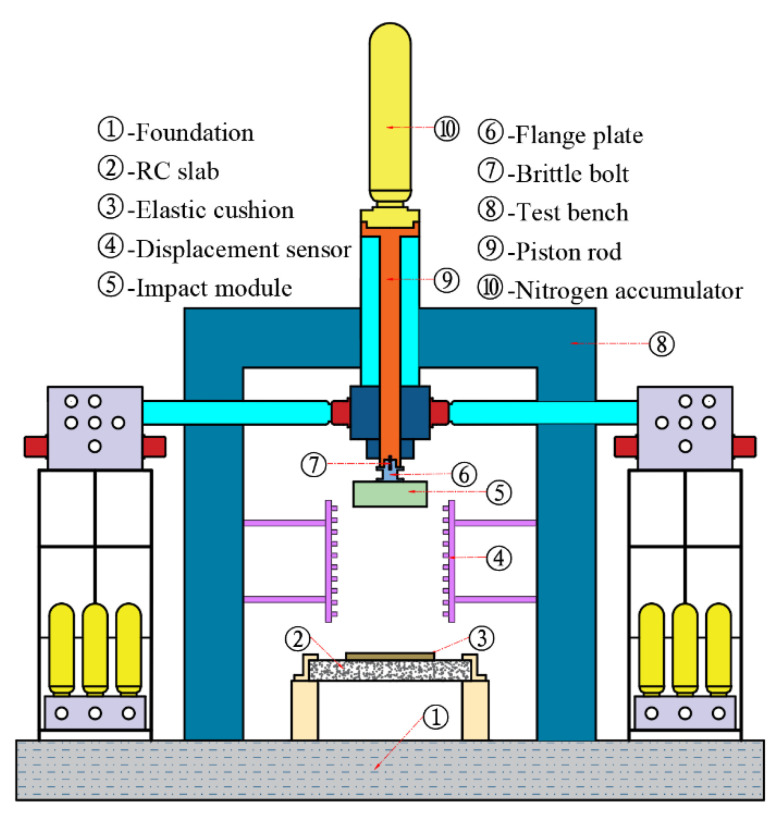
Diagram of the VMLH Blast Simulator.

A new impact-based facility has been developed that vertically fires multi-mass impact modules to load large components at precise high velocities (VMLH). The VMLH Blast Simulator is powered by a high-speed gas-hydraulic actuator, as shown in Figure 1. Compared to traditional chemical explosion tests, this equipment has the advantage of easy data acquisition, high reproducibility and reliability, especially without the fireball generated by the explosion, and the ability to use high-speed cameras to capture the deformation–destruction evolution of the component.

Before the test, the nitrogen cylinder is inflated and stored. During the test, the high pressure nitrogen is released instantaneously, and the piston rod and impact module connected by the brittle bolt are accelerated together. Then, during the acceleration process, a number of high-speed switching valves in the hydraulic system are opened simultaneously, and the oil in the impact cylinder flows back rapidly; when the impact module reaches the set speed value, the oil pressure rises, producing a buffering effect on the piston rod to slow down. At this point, the brittle bolt breaks, and the impact module, which is separated from the piston rod, starts a short free fall movement until it hits the specimen vertically. The test process is shown in Table 1.

The VMLH Blast Simulator is designed to simulate the effects of an explosion on a component or structure. Similar to other blast simulators [12,17], it achieves this by creating non-elastic collisions between the impact module and the specimen. This is carried out by adding a shock-absorbing layer between them, which transmits the load to a reinforced concrete slab in the form of waves. In this article, silicone rubber is arranged between the impact module and the specimen to adjust the shape of the force during the collision. When the impact module falls on the rubber, the rubber undergoes elastic deformation, producing lateral waves or shear waves. These waves propagate along the rubber until they reach the edge of the reinforced concrete slab and are reflected back. The rubber also produces longitudinal waves or compression waves, which propagate through the reinforced concrete slab, reach the lower surface of the slab, and are reflected back. These waves cause vibration and deformation of the slab. When the explosion load is applied to the component, a pressure wave called the explosion wave will be generated. This pressure wave will propagate inside the component and the surrounding medium, causing damage to the component and the surrounding environment. Under the action of explosion load, the stress and deformation inside the component will undergo drastic changes, which may lead to the fracture, deformation or collapse of the component.

### 2.2. Test Specimen

**Figure 2:**
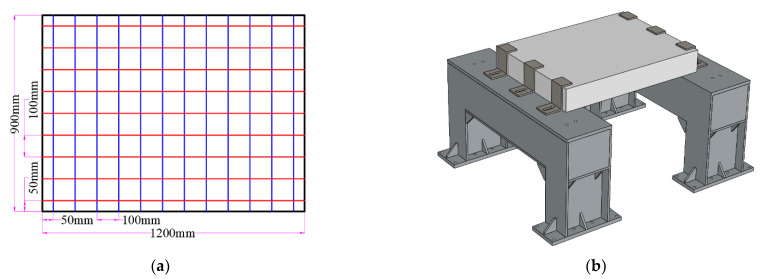
Preparation of RC slabs. (**a**) Dimensions and reinforcement; and (**b**) supporting structure.

The dimension of these slabs are 1200 mm length, 900 mm width and 180 mm depth. They are reinforced in both directions with a 10 mm steel bar reinforcement mesh with 100 mm spacing and 25 mm of concrete cover. All the RC slabs were cast from the same batch of commercial concrete with a strength class of C40. Five 150 mm cubes were cast and tested. The average strength of the concrete cubes after 28 days of curing is 45.6 MPa. The type of reinforcement is HRB400E, which has a yield strength of 435 Mpa and a Young’s modulus of 209 Gpa. The dimensions of the slab, in addition to the reinforcement detailing and supporting conditions, are shown in Figure 2.

### 2.3. Impact Cushion

**Figure 3:**
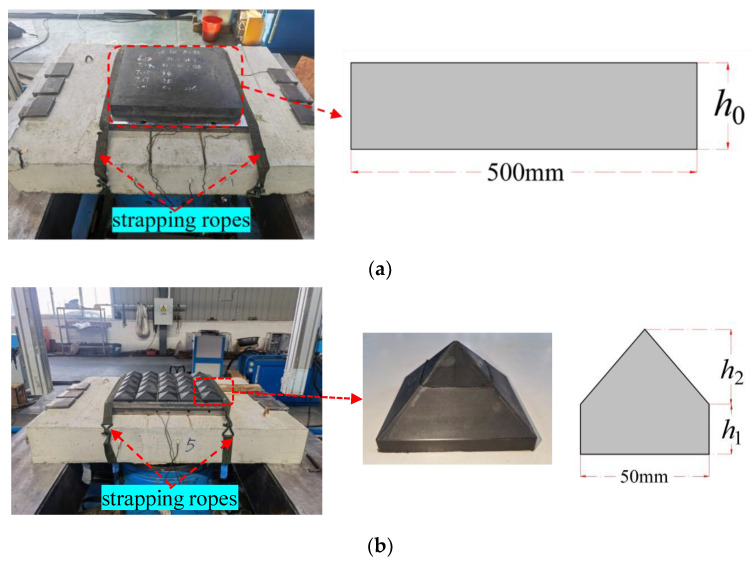
Impact cushion and installation. (**a**) Planar rubber; and (**b**) pyramidal rubber.

Silicone rubber (referred to as rubber for short) is well-established, inexpensive to prepare, and can be reused in trials. The silicone rubber is placed on the surface of the specimen. On one hand, it prevents direct contact between the impact module and the specimen, which may cause damage to the metal material. On the other hand, the existence of the rubber achieves flexible contact with the specimen, avoiding small angle deviations that may occur during the falling process of the impact module, which may cause uneven loading of the specimen. More importantly, the viscoelastic material properties of the rubber determine the waveform of the load transmitted by the impact. Therefore, rubber was chosen as the impact cushion, and two shapes were designed, as shown in Figure 3. The planar rubber size is 500 mm × 500 mm with a thickness range of 20~100 mm. The pyramidal rubber size is 100 mm × 100 mm, and the height is 50 mm, where the values of bottom thickness *h*_1_ and upper thickness *h*_2_ are 20 mm and 30 mm, respectively.

### 2.4. Experimental Setup

**Figure 4:**
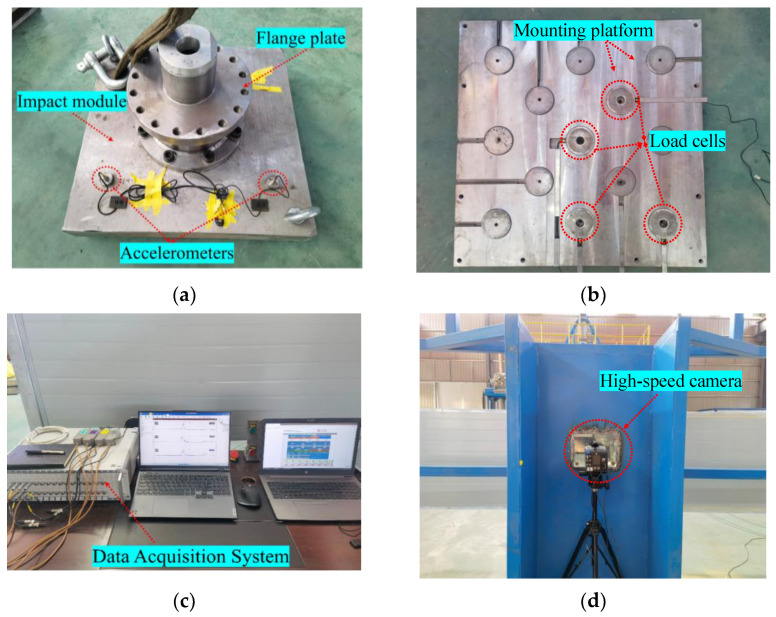
Composition of the measurement system. (**a**) Accelerometer sensors; (**b**) Impact force sensors; (**c**) Data acquisition system; and (**d**) High-speed cameras.

A set of displacement sensors was used to measure the impact module speed, as shown in Figure 1. Two accelerometers (SD1407) were attached to the impact module for measuring acceleration, with a range of 5000 g and a sensitivity of 2.2~2.64 pc/g, as shown in Figure 4a. Four load cells (KD3050) were used to measure the impact forces, with a range of 5000 g and a sensitivity of 19.6~19.88 mv/kN, as shown in Figure 4b. These were placed in the holes of the mounting platform, followed by the cover plate and pre-tightened with a screw. To prevent the mounting platform from oscillating significantly during the test, it was secured using two strapping ropes, as shown in Figure 3.

The test data were acquired using the super dynamic signal test and analysis system (DH5960), and the PCO high-speed camera was used to record the impact module from the acceleration to impact with the specimen mass, as shown in Figure 4c. The data acquisition system was set up with six channels, two of which were accelerometers and four were load cells, and the sampling rate was set to 500 kHz. The high-speed camera was connected to a computer with the Camware4 commercial software installed to manage the recording process, as shown in Figure 4d. The high-speed camera used in this test has a frame rate of up to 4500 fps and a resolution of 680 × 1200 pixels.

## 3. Experimental Results and Discussion

The impact tests were carried out using a 100 kg impact module with an adjustable impact speed and impact cushion to apply a range of impact loading to the specimens. A total of nine tests were carried out on four specimens. For tests 1 to 5, planar rubber thicknesses ranging from 20 to 100 mm, and impact velocities of approximately 15 m/s were selected. Tests 6 to 9 used pyramidal rubber in a 5 × 5 arrangement, with impact velocities of 10 to 25 m/s. Tests 10 and 11 were repeat control tests of Test 3. Table 2 summarizes the impact velocity, contact force and loading time for each test and provides the results for the pressure and shock volumes. The calculation of the parameters and the analysis of the test results are presented below.

**Figure 5:**
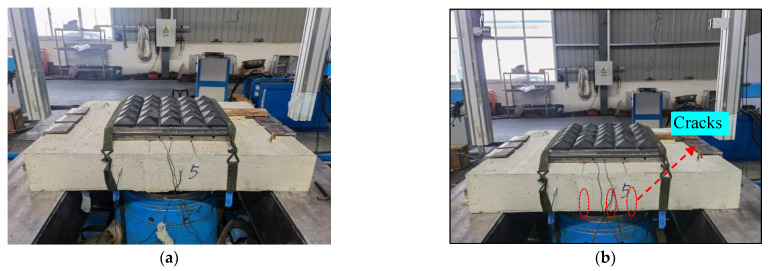
Damage condition of the specimen after a single impact. (**a**) Before the test; and (**b**) After the test.

Based on the assumption of uniform loading, we do not want the specimen to experience significant deformation and damage, as this would not be conducive to the repeatability of our measurement results. As you can see, this is reflected in the test speed, which is controlled below 25 m/s, far below the equipment’s speed limit, making the specimen less prone to damage. Figure 5 shows the damage to the specimen before and after a single impact, with several cracks appearing but with no significant deformation occurring. Due to the distance of the specimen’s bottom from the buffer bar being only about 100 mm, it was not possible to effectively place the displacement sensors to obtain the mid-span deflection value of the specimen.

### 3.1. Methodologies for Calculated Parameters

#### 3.1.1. Load

**Figure 6:**
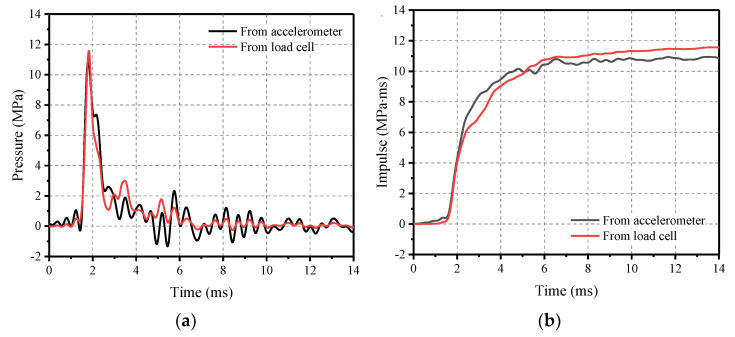
Impact loading measured using accelerometers and load cells. (**a**) Pressure profile; and (**b**) Impulse profile.

The accelerometers and load cells were strategically placed to capture test data, which was then used to calculate pressure and impulse. Assuming that the VMLH Blast Simulator applies a uniform load to the surface of the specimen, the accelerometer data was converted to force by multiplying it with the weight of the impact module, and to pressure by dividing it by the impact area. Similarly, load cell data was converted directly to pressure by dividing it by the impact area. For instance, the pressure and impulse data obtained from Test 8 are illustrated in Figure 6. The impact loads obtained from both methods were found to be very similar, which is why the load cell data was primarily used for subsequent analysis.

#### 3.1.2. Velocity

**Figure 7:**
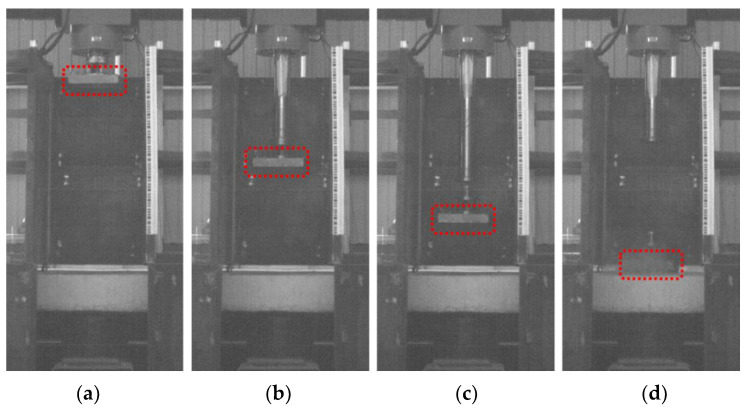
High-speed video of impact test. (**a**) Inflation; (**b**) Acceleration; (**c**) Separation; and (**d**) Impact.

A high-speed camera was used to record the impact test procedure. Figure 7 illustrates the four distinct stages of compression, acceleration, separation, and impact. Following the separation from the brittle bolt, the impacting module is still 0.9 m away from the specimen surface and begins to fall freely. As a result, the velocity of the impact module at the moment of impact with the specimen can be determined by calculating the final velocity of the displacement sensor.

### 3.2. Comparison of Impact Loading and Blast Loading

**Figure 8:**
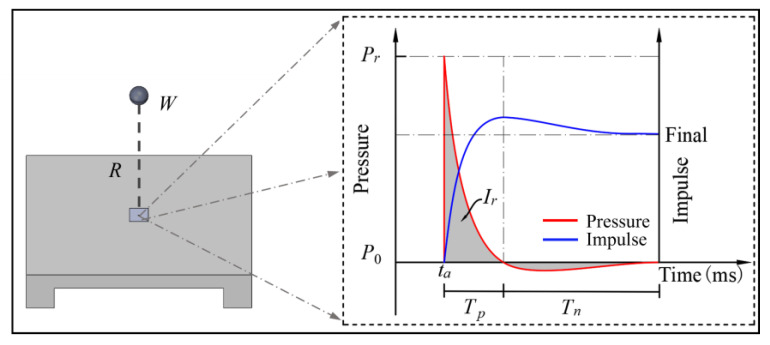
Blast scenario with representative pressure profile.

A typical blast scenario is shown in Figure 8, which includes a spherical charge of TNT weight, W, at a standoff distance, R, away from a structure [19,20]. The detonation of the explosive creates a shock wave that forms a reflected wave when it reaches the surface of the structure. Under these conditions, an example of a typical reflected pressure profile at a point on the structure is also shown in Figure 8, where *P_r_* is the peak reflected overpressure, and *T_p_* is the positive phase duration. The area under the pressure–time history is the specific impulse (hereafter simply referred to as impulse). As the value of the negative pressure is much smaller than the positive pressure [21], this study will only focus on the positive phase of the impulse, *I_r_*. Various methods have been used to evaluate the true values of *P_r_*, *T_p_* and *I_r_* [22].

**Figure 9:**
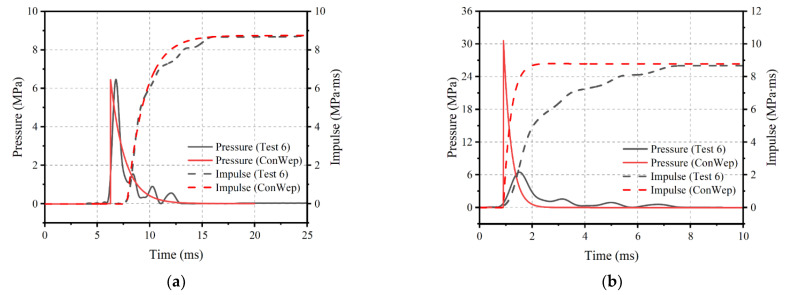
Comparison of impact loading and ideal blast loading. (**a**) Pressure–impulse criterion; (**b**) Impulse criterion.

Close-in charges, such as roadside car bombs, last between 2 and 4 ms and have an impulse maximum of about 11 Mpa∙ms to 15 Mpa∙ms [19,23,24]. As long as the characteristic response time of the specimen is greater than four times the duration of the impulse, the impulse will dominate the response of the specimen, regardless of the exact shape of the pressure–time history [25]. Civil structures, including individual elements, such as beams and slabs, meet this condition. Therefore, this paper discusses two equivalence criteria for simulating blast loading, one that considers only the impulse force without regard to the exact shape of the pressure curve, and the other that considers both the impulse and the pressure–time curves. An example of equivalent conversions for Test 4 is given in the Table 3, where the parameters of blast environment have been obtained using graphical methods in TM5-1300 [24]. The data in the TM5-1300 manual is based on real test data and empirical formulas, and has been verified and applied multiple times, and widely cited in a series of studies [26,27,28,29]. In Figure 9, the pressure–time history of Test 6 is compared to the corresponding ideal blast profile, which is calculated using ConWep [30]. The ConWep algorithm is an empirical formula for calculating explosive loads. By inputting parameters, such as the type, mass, initiation method, distance, and height of the explosive, various aspects of the explosive load can be calculated [31]. The pressure–impulse criterion is used to evaluate the blast loading, and it is found that the blast loading closely matches the pressure and impulse of the impact loading. This comparison shows that both equivalence criteria are suitable for simulating blast loading. However, when using the pressure–impulse criterion for conversion, the resulting impact is equivalent to a blast condition; when using the impulse criterion, the results are not unique and the charge must be assumed before the corresponding blast parameters can be calculated.

### 3.3. Analysis and Discussion

**Figure 10:**
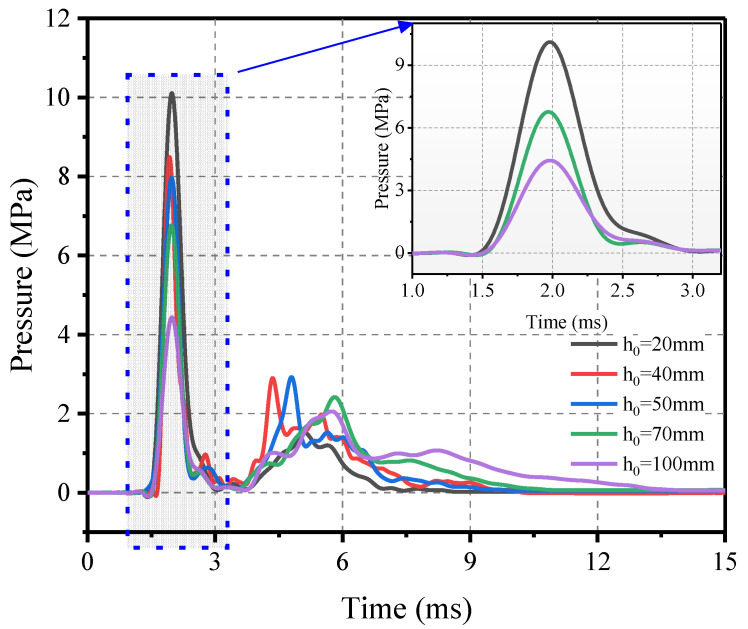
Pressure–time history versus thickness.

The results of tests 1 to 5 are shown in Figure 10. As the thickness of the planar rubber sheet increases, the peak pressure decreases and the impulse also tends to increase gradually. However, there is a significant difference in the curve profile between the impact loading and the ideal blast loading. For example, at a cushion thickness of 50 mm, the impact loading first rises rapidly, then falls rapidly to zero, then rises again to around 2 MPa and finally falls slowly. This phenomenon is due to the oscillations of the rubber. The phenomenon of secondary peaks may be related to the compression of the rubber. After the initial contact, the rubber is compressed and removed from the specimen surface, at which point the contact force is almost zero; as the rubber reaches the densification stage, the load increases again and the curve becomes smoother. Therefore, the shock loads generated under the above conditions cannot be converted to blast loading using the pressure–impulse criterion, thus, the impulse criterion should be used.

**Figure 11:**
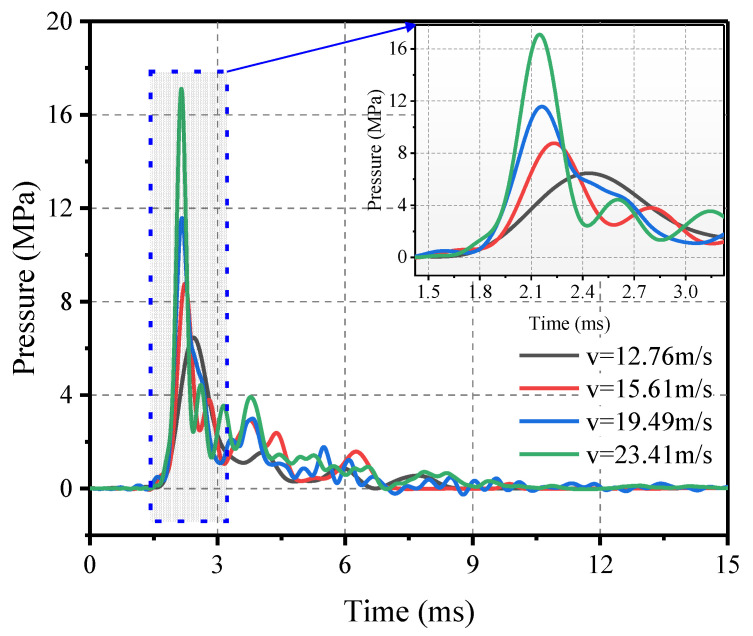
Pressure–time history versus impact velocity.

The results of tests 6 to 9 are shown in Figure 11. The shape of the pressure profile is characterized by a steep increase in pressure followed by a rapid decay for a duration of approximately 3 to 5 s, which is normal for the equivalent blast loading. From the peak trend, it is evident that pressure and impulse increase as the shock velocity increases. The impact loading is smoother due to the pyramidal shape of the rubber. Compared to the planar rubber case, the pressure tends to fall more gently after reaching its peak, although the difference is not significant initially. This makes it possible to apply both equivalence criteria to simulate blast loading when pyramidal rubber is used as the impact cushion.

**Figure 12:**
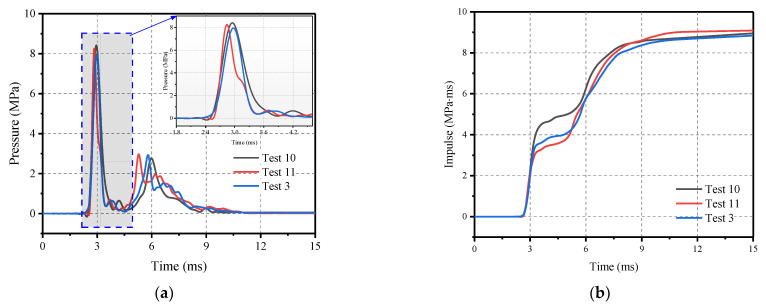
Comparison of impact loading of three repeated tests (**a**) Pressure–time curve; and (**b**) Impulse–time curve.

Table 4 presents the impact force, loading time, pressure, and impulse for three repeated tests, along with the average and variance of the data. In addition, Figure 12 shows the pressure and impulse time history curves of the three sets of repeated experiments. The results indicate that the equipment has high loading accuracy and the data collection reliability of the test is also high, meeting the requirements of load repeatability for mechanical impact simulation explosion tests.

## 4. Numerical Simulations

In this study, we utilized the non-linear dynamic analysis software LS-DYAN to simulate the impact loading caused by RC plates when subjected to a blast simulator. Through a comparison of the numerical simulations and experimental test data, we were able to verify the accuracy of the numerical model and the reliability of the test method. Additionally, we investigated the effect of rubber shape, the impact velocity, the bottom thickness, and the upper thickness on the impact loading.

### 4.1. Material Models

#### 4.1.1. Concrete

The CSCM CONCRETE (MAT_159) material model, which is available in LS-DYNA, is used to simulate the dynamic performance of reinforced concrete protection structures during vehicle collisions [32]. This material model was developed by the Federal Highway Administration and its parameters are defined based on the results of cubic compression tests. Table 5 shows the parameters of this material model that are used in the present study to model concrete. It is important to note that this material model is specifically designed to simulate the behavior of roadside reinforced concrete protection structures and has been validated for this purpose.

#### 4.1.2. Steel

The steel of the slabs in the present study is modeled using the material model Plastic Kinematic (MAT_003) in Ls-Dyna [33], which is an elastic-plastic model with kinematic and isotropic hardening. Reports of material property tests provided by steel producers are used in the numerical simulation of test cases. The expression for the dynamic yield strength of steel, taking into account the effect of strain rate on the intrinsic structure relationship of the material, is as follows:

(1) $$\sigma_{y}=\left[1+{\left(\dot{\varepsilon }/C\right)}^{1/P}\right]\left(\sigma_{0}+\beta E_{P}\varepsilon_{P}{}^{eff}\right)$$

where, $\sigma_{y}$ is the dynamic yield strength of the steel, $\dot{\varepsilon }$ is the strain rate, *C* and *P* are the parameters of the strain rate, $\sigma_{0}$ is the initial yield strength of the steel, *β* is the hardening parameter, $E_{P}$ is the hardening modulus, and $\varepsilon_{P}{}^{eff}$ is the effective plastic strain. The input material parameters of steel in the current study are tabulated in Table 5. The Plastic Kinematic material model in Ls-Dyna is capable of accurately capturing the complex material behavior of steel under such extreme loading conditions.

#### 4.1.3. Rubber

Blatz–Ko rubber is a combination of Blatz and Ko [34] defined by a hyper-elastic rubber model using type II Piola–Kirchoff stresses. The Blatz–Ko strain energy density function is a powerful tool for modeling compressible types of rubber, and it can be expressed in a precise mathematical form.

(2) $$W=\frac{1}{2}G\left(\frac{I_{2}}{I_{3}}+2\sqrt{I_{3}}-5\right)$$

where, *G* is the shear modulus at infinitesimal deformation, *E* is the Young’s modulus of elasticity and υ is the Poisson’s ratio. $I\left(n=1,2,3\right)$ is the invariant of the Cauchy–Green deformation tensor. Equation (2) contains only one material constant, *G*. The material parameters are shown in Table 5.

**Table 5:** Input parameters for concrete, steel and rubber material models.

Material	Parameter	Value	Comments
Concrete	RO (Density)	2400 kg/m^3^	Material test data
	FPC (Uniaxial compression strength)	45.6 MPa	
	NPLOT	1	According to [32,35,36]
	INCRE	0	
	IRATE (Rate effects options)	1	
	Elements erode	1.1	
	RECOV	0	
	IRETRC (Cap retraction option)	0	
	Pre-existing damage	0	
	DAGG (Maximum aggregate size)	24 mm	
	UNITS (Units options)	4	
Steel	Density	7800 kg/m^3^	Material test data
	Young’s modulus	2.09 × 105 MPa	
	Poisson’s ratio	0.3	
	Yield stress	435.3 or 450.1 MPa	
Rubber	Density	1.27 kg/m^3^	According to [34,37]
	Poisson’s ratio	0.463	
	Shear modulus	24 MPa	

### 4.2. Model Calibration and Validation

#### 4.2.1. Numerical Model

**Figure 13:**
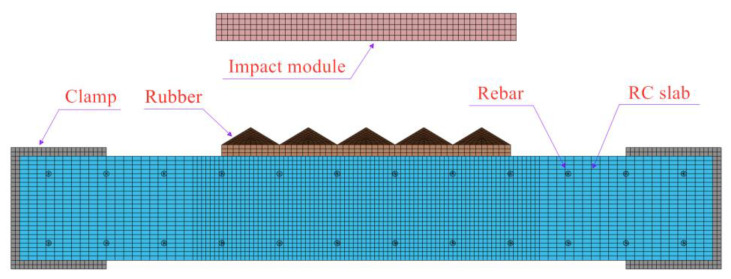
Finite element model.

The test results clearly indicate that using flat rubber as an impact cushion leads to a secondary peak in the impact load, which is distinct from blast loading and thus unsuitable for simulating them. Therefore, a parametric analysis of pyramidal rubber was conducted to investigate the impact loading characteristics, taking into account the effects of impact velocity and rubber thickness. Figure 13 illustrates the numerical model used in this study. Initially, the original design was a 500 mm long and 500 mm wide pyramidal rubber during the experiment, which required a large mold for processing. Considering the cost and processing time, it was divided into 25 small pyramidal rubbers measuring 100 mm in length and width, each requiring only a small mold. In the numerical calculations, since the dispersed small pyramidal shapes needed to be considered for contact, we simplified the model accordingly. Based on the comparison between the numerical and experimental results, this simplification was found to be feasible.

**Figure 14:**
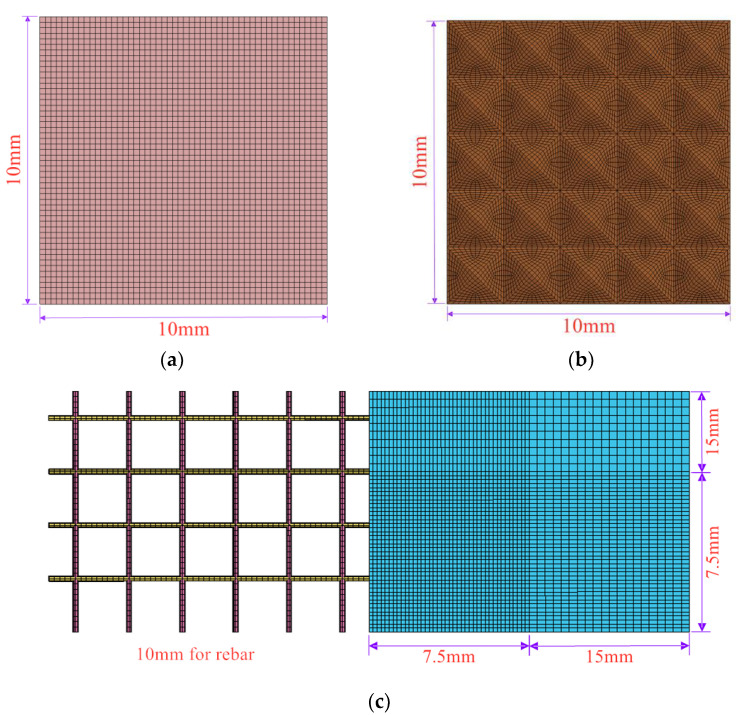
Detail of the grid division. (**a**) Impact module; (**b**) Rubber; (**c**) and RC slab.

The model comprises an RC plate, rubber, steel impact module, and fixture. Meshing was performed using 8-node solid hexahedral cells, and mesh convergence analysis was conducted to determine the appropriate cell size. Based on the convergence analysis, a concrete mesh size of 7.5 mm was used, which was doubled outside the range of ±600 mm from the center of the slab to reduce calculation time. The impact module had a cell size of 10 mm in both the side length and thickness direction. The upper half of the rubber was pyramidal and tangentially treated to achieve a mesh size of approximately 10 mm. The grid division details are presented in Figure 14. Further mesh refinement was found to yield similar simulation results, but would significantly increase calculation time. Details of the mesh refinement analysis will be presented in Section 4.2.3.

#### 4.2.2. Boundary Conditions

Reasonable boundary conditions are crucial for obtaining accurate numerical results. In this study, we assume the supporting structure of the RC slab to be a rigid body that is fixed, and thus surface-to-surface contact was used, which uses a penalty function to determine the contact force to define the contact between the specimens and the supporting structure to constrain the specimens. To simplify the calculation and save computing resources, we directly define the impact velocity using *INITIAL_VELOCITY_GENERATION, applying a downward vertical initial velocity to the impact module. Additionally, a surface-to-surface contact was used to define the contact between the impact module, rubber, and concrete.

#### 4.2.3. Grid Refinement Analysis

The numerical model’s grid size was determined by conducting five analyses with varying grid resolutions, and the results are presented in Table 6. The grid convergence tests involved five cell sizes, namely 5 mm, 7.5 mm, 10 mm, 20 mm, and 30 mm. The peak pressures calculated for the models with the five grid sizes were found to be very similar, with a maximum error of only 3.41%. This indicates that reducing the grid size has little impact on the numerical results, but it significantly increases the computational time. Therefore, a grid size of 7.5 mm was selected for this study to strike a balance between accuracy and computational efficiency.

#### 4.2.4. Comparison of Experimental and Numerical Results

**Figure 15:**
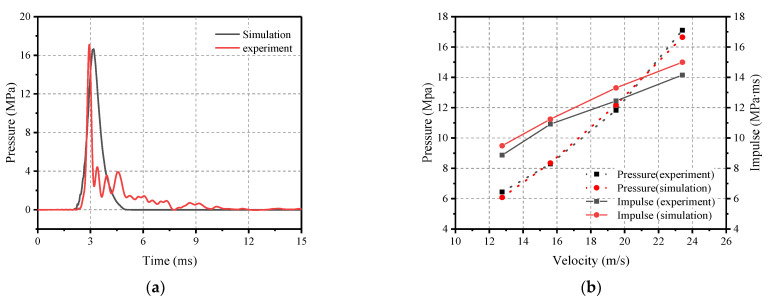
Comparison of numerical and experimental results. (**a**) Pressure profile; and (**b**) Peak pressure and impulse.

The numerical model was calibrated by comparing the results of numerical calculations with the experimental test results. In Figure 15a,b, the pressure–time histories of Test 9 and the peak pressure and impulse of Tests 6 to 9 are presented, respectively. Additionally, the percentage differences between the experimental results and the numerical results are presented in Table 7. The comparison results indicate that the pressure curve obtained via numerical simulation is in good agreement with the measurement results.

### 4.3. Parametric Studies

#### 4.3.1. Effect of Rubber Shapes

**Figure 16:**
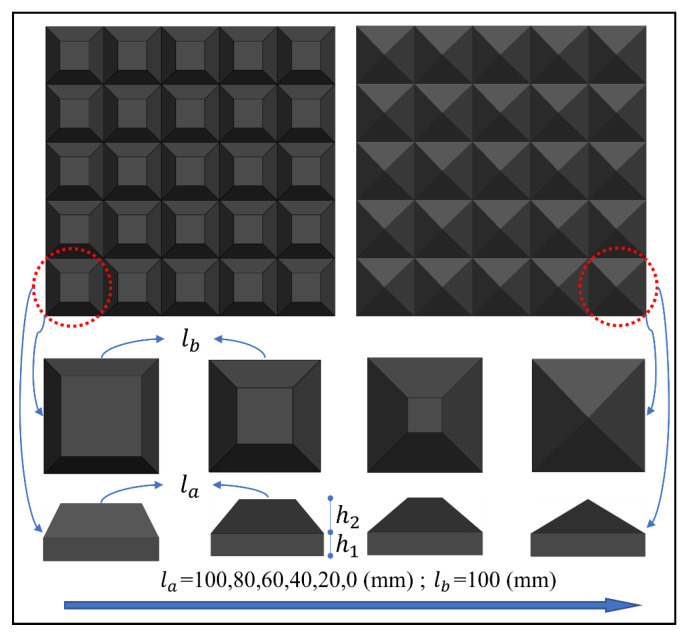
Schematic of rubber shapes variation of the upper side.

**Figure 17:**
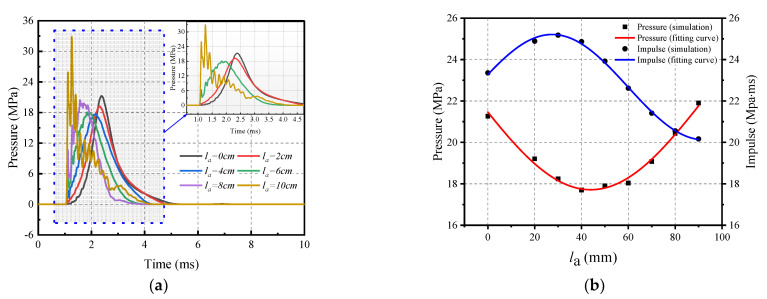
Influence of rubber shapes on the impact loading. (**a**) Pressure–time history; and (**b**) Peak pressure and impulse.

The effect of rubber shape on the impact loading was investigated by comparing the results of six rubber shapes that are shown in Figure 16. The impact velocity was 20 m/s and the values of *h*_1_ and *h*_2_ are 20 mm and 30 mm, respectively. Figure 17a shows the impact response of six rubber shapes of rubber as an impact cushion for a reinforced concrete slab with a thickness of 180 mm. As expected, the pressure–time curve is smoothest when the upper side *l_a_* is equal to 0. As *l_a_* gradually increases, the pressure–time curve begins to oscillate and reaches a maximum when *l_a_* is 100 mm. In addition, Figure 15b, highlights the peak pressure and impulse of the impact response for *l_a_* from 0 to 90 mm and the fitted curve, excluding the case where the upper side *l_a_* is 100 mm. The peak pressure and impulse of the upper side between 0 and 90 mm can be calculated according to the following equation.

(3) $$P_{l}{}_{{}_{a}}=22.124+4.409\times \sin\left(\pi \times \left(l_{a}-9.254\right)/9.72\right),\;0\leq l_{a}\leq 90\;\mathrm{mm}$$

(4) $$I_{l}{}_{{}_{a}}=22.668+2.543\times \sin\left(\pi \times \left(l_{a}+0.498\right)/6.49\right)\;,0\leq l_{a}\leq 90\;\mathrm{mm}$$

#### 4.3.2. Effect of Impact Velocities

**Figure 18:**
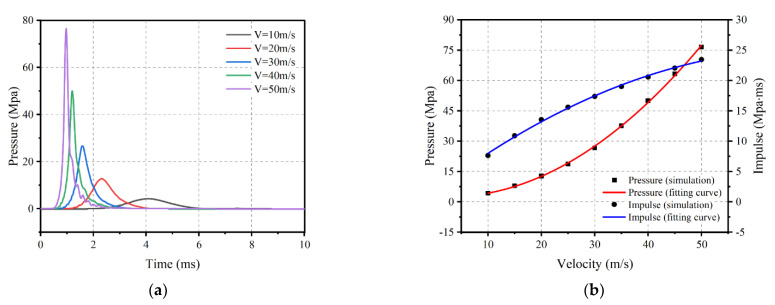
Influence of impact velocity on the impact loading. (**a**) Pressure–time history; and (**b**) Peak pressure and impulse.

To further characterize the effect of velocity on the impact loading characteristics, velocities ranging from 10 to 50 m/s were set, and numerical calculations were carried out. Figure 18 shows the load profile and the relationship between peak pressure and impulse versus velocity. As the speed increases, the peak pressure gradually increases, while the time of the load decreases accordingly, a connection can be established using Equation (5). The impulse also increases with velocity, unlike the pressure, which increases at a progressively slower rate and can be described using Equation (6).

(5) $$P_{v}=-21.275+17.135e^{-v/28.489}$$

(6) $$I_{v}=565.256-13.415e^{\left(-v/9.804\right)}-555.419e^{\left(-v/1972.268\right)}$$

#### 4.3.3. Effect of Bottom Thicknesses

**Figure 19:**
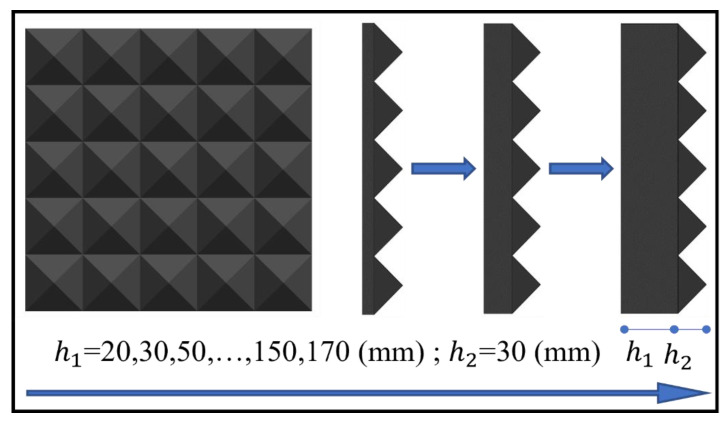
Schematic of the variation of the rubber bottom thickness *h*_1_.

**Figure 20:**
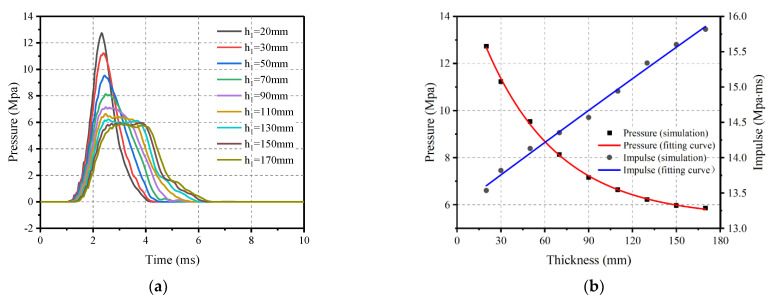
Influence of bottom thickness ***h***_1_ on the impact loading. (**a**) Pressure–time history; and (**b**) Peak pressure and impulse.

The effect of bottom thickness *h*_1_ on the impact loading was investigated by comparing the results of ten bottom thicknesses. At an impact velocity of 20 m/s, the *h*_2_ value was held constant at 30 mm while the *h*_1_ varied from 20 mm to 170 mm, as illustrated in Figure 19. The peak pressure initially decreases significantly as *h*_1_ increases, and then gradually decreases while impulse increases linearly with the *h*_1_, as shown in Figure 20. To simplify the calculations, the results are fitted with the peak pressure calculated as shown in Equation (7) and with the impulse calculated using Equation (8).

(7) $$P_{h_{1}}=5.454+10.794e^{-h_{1}/4.983},\;2\;\mathrm{cm}\leq h_{1}\leq 17\;\mathrm{cm}$$

(8) $$I_{h_{1}}=61.479-48.185e^{-h_{1}/310.853},\;2\;\mathrm{cm}\leq h_{1}\leq 17\;\mathrm{cm}$$

#### 4.3.4. Effect of Upper Thicknesses

**Figure 21:**
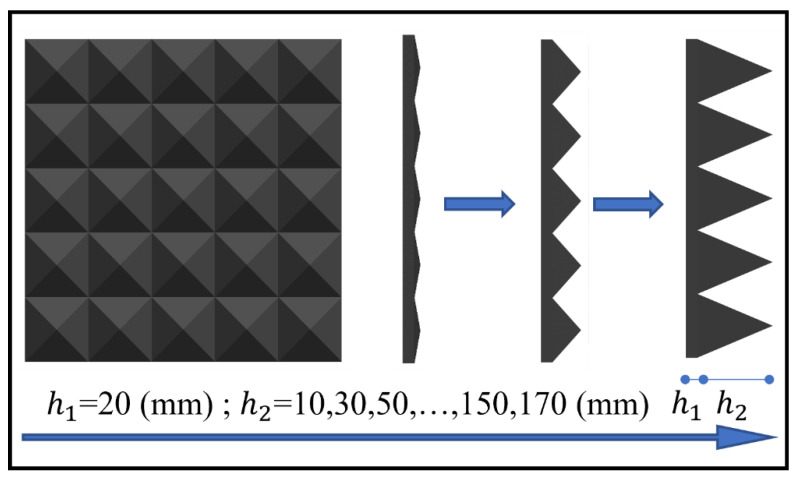
Schematic of the variation of the rubber upper thickness *h*_2_.

**Figure 22:**
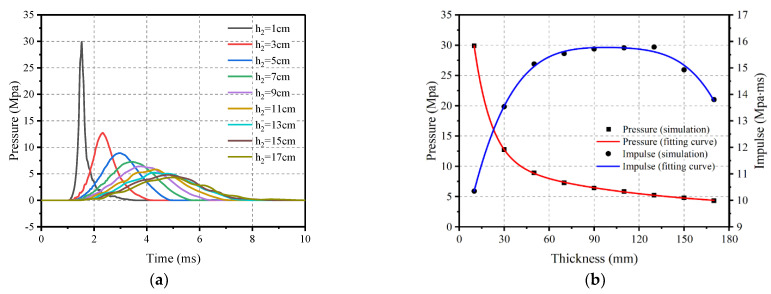
Influence of upper thickness *h*_2_ on the impact loading. (**a**) Pressure–time history; (**b**) Peak pressure and impulse.

Similarly, the effect of upper thickness on the impact loading was investigated by comparing the results of nine upper thicknesses of pyramidal rubber. At an impact velocity of 20 m/s, the bottom thickness *h*_1_ was held constant at 20 mm while the *h*_2_ thickness varied from 10 mm to 130 mm, as detailed in Figure 21. The impact response of nine upper thicknesses of rubber as impact cushion for a reinforced concrete slab with a thickness of 180 mm is shown in Figure 22.

It can be seen that the pressure gradually decreases while the time of load and impulse increase accordingly as *h*_2_ gradually increases. It is worth noting that when *h*_2_ is between 70 mm and 130 mm, the impulse is almost unaffected, accompanied by a significant reduction as *h*_2_ continues to increase. To simplify the calculations, the results are fitted with the peak pressure calculated as shown in Equation (9) and with the impulse calculated using Equation (10).

(9) $$P_{h}{}_{{}_{2}}=2.664+45.495e^{-h_{2}/1.14}+9.17e^{-h_{2}/10.12},\;1\;\mathrm{cm}\leq h_{2}\leq 17\;\mathrm{cm}$$

(10) $$I_{h}{}_{{}_{2}}=7.811-2.927\times h_{2}-0.41\times h_{2}{}^{2}+0.026\times h_{2}{}^{3}-6.441^{-4}\times h_{2}{}^{4},\;1\;\mathrm{cm}\leq h_{2}\leq 13\;\mathrm{cm}$$

## 5. Summary and Conclusions

In this study, experimental tests were performed on four specimens to demonstrate the feasibility of the VMLH Blast Simulator for simulating blast loading. A numerical model was also developed to predict the impact loading using LS-DYNA. The validity of the model has been calibrated against experimental test results. Using the calibrated model, further studies are carried out to investigate the effect of different parameters on the impact loading of RC plates. The parameters investigated within the scope of this study were rubber shapes, impact velocities and the bottom thicknesses and the top thicknesses of the pyramidal rubber. The following conclusions can be drawn from the detailed experimental and numerical studies presented in this paper.

(1) The use of pyramidal rubber with a 0 mm upper side was more effective in regulating the peak pressure and impulse of impact loading compared to planar rubber with a 100 mm upper side. This was evident in the pressure–time curve, which closely resembled ideal blast loading. However, when *l_a_* was between 40 mm and 100 mm, the pressure profile oscillated significantly, making it unsuitable for the pressure–impulse criterion.

(2) The impact velocity was found to have a significant effect on the pressure and impulse of impact loading. For a pyramidal rubber thickness of 50 mm, both pressure and impulse increased rapidly with increasing velocity. When the speed increases from 12.76 m/s to 23.41 m/s, the corresponding range of peak pressure is from 6.457 to 17.108 MPa, with an increase of 164.22%. The corresponding range of impulse is from 8.573 to 14.151 MPa∙ms, with an increase of 65.07%.

(3) Variations in the upper thickness of the pyramidal rubber have a more positive effect on the impact loading than the bottom thickness. Notably, increasing the top thickness from 30 mm to 130 mm resulted in a 59.01% decrease in peak pressure and a 16.64% increase in impulse. Conversely, increasing the bottom thickness from 30 mm to 130 mm resulted in a 44.59% decrease in peak pressure and an 11.01% increase in impulse. As the bottom thickness increases, it takes longer for the rubber to compress and become compressed. When bottom thickness is 110 mm, the pressure reaches its peak and then barely changes in a time of approximately 2 ms, which is unsuitable for using the pressure–impulse criterion for modeling blast loading. Increasing the upper thickness only allows the pressure to rise and fall more smoothly without changing its shape characteristics, making it possible to adopt both criteria for simulating blast loading.

(4) The impact loading and blast loading can be converted using the “pressure-impulse” and “ impulse” criterion. By obtaining the peak pressure and impulse of the impact loading, a corresponding explosive environment can always be found. These criteria can be widely applied in simulating blast loading using non-explosive methods.

## Figures and Tables

**Table 1:** Test procedure of the VMLH Blast Simulator.

t=0	$0<t<t_{i}^{-}$	$t=t_{i}^{-}$	$t=t_{i}$
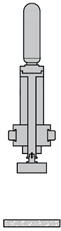	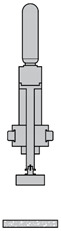	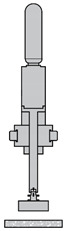	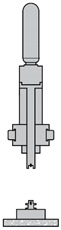
Inflation	Acceleration	Separation	Impact

**Table 2:** Experimental results (force, time, peak pressure, and impulse).

Tests No.	Specimen No.	*h* (mm)	*v* (m/s)	*F* (kN)	*T* (ms)	*P* (MPa)	*I* (MPa∙ms)
1	1	20	15.58	2526.507	6.034	10.106	8.482
2	1	40	15.56	2121.595	7.801	8.486	8.720
3	2	50	15.56	1991.483	8.164	7.966	9.119
4	2	70	15.61	1690.673	9.166	6.763	9.755
5	2	100	15.63	1108.908	12.326	4.436	10.452
6	3	50	12.76	1611.070	6.848	6.457	8.573
7	3	50	15.61	2072.210	5.594	8.766	10.402
8	3	50	19.49	2894.249	5.481	11.577	11.915
9	4	50	23.41	4277.063	5.322	17.108	14.151
10	5	50	15.53	2103.832	7.862	8.415	9.779
11	5	50	15.65	2064.585	8.258	8.258	9.224

Notes: *h*—Planar rubber thickness; *v*—Velocity; *F*—Force; *T*—Time; *P*—Peak pressure; *I*—Impulse.

**Table 3:** Equivalent conversion of impact loading and blast loading.

*Q*	Impact Environment			Explosive Environment				
	*v* m/s	*P* MPa	*I* MPa∙ms	*W* kg	*R* m	*Z* m/kg^1/3^	*P* MPa	*I* MPa∙ms
①	12.76	6.457	8.869	2690.679	12.728	0.915	6.457	8.869
②	12.76	6.457	8.869	216	2.964	0.494	31.206	8.869

Note: *Q*—Criteria for equivalence; *v*—Velocity; *T*—Time; *P*—Peak pressure; *I*—Impulse; *W*—Spherical charge of weight; *R*—Standoff distance; *Z*—Scaled distance; ①—Pressure–impulse criterion; ②—Impulse criterion.

**Table 4:** Experimental results of three repeated tests.

Tests No.	*F* (kN)	*T* (ms)	*P* (MPa)	*I* (MPa∙ms)
3	1991.483	8.164	7.966	9.119
10	2103.832	7.862	8.415	9.779
11	2064.585	8.258	8.258	9.224
Average	2053.3	8.095	8.213	9.374
Variance	2167.392	0.029	0.035	0.084

Notes: *F*—Force; *T*—Time; *P*—Peak pressure; *I*—Impulse.

**Table 6:** Mesh sizes and results for mesh refinement analysis.

Unit Size (mm)	5	7.5	10	20	30
Pressure (MPa)	26.420	26.505	26.107	25.934	25.631
Impulse (MPa∙ms)	17.058	17.358	17.445	17.322	17.203
Computational time (min)	34	13	7	2	1

**Table 7:** Comparison of numerical calculations with experimental test.

Tests No.	Experimental Test		Simulation		Deviation	
	Pressure (MPa)	Impulse (MPa∙ms)	Pressure (MPa)	Impulse (MPa∙ms)	Pressure (%)	Impulse (%)
6	6.457	8.870	6.083	9.489	−5.602%	6.979%
7	8.289	10.912	8.35	11.242	0.736%	3.024%
8	11.577	11.912	12.18	13.307	5.209%	11.711%
9	17.108	14.152	16.646	14.997	−2.700%	5.971%

## Data Availability

The data presented in this study are available on request from the corresponding author.

## References

[1] Liao Q., Yu J., Xie X., Ye J., Jiang F. (2022). Experimental study of reinforced UHDC-UHPC panels under close-in blast loading. J. Build. Eng..

[2] Wang L., Cheng S., Liao Z., Yin W., Liu K., Ma L., Wang T., Zhang D. (2022). Blast Resistance of Reinforced Concrete Slabs Based on Residual Load-Bearing Capacity. Materials.

[3] Wang W., Yang G., Yang J., Wang J., Wang X. (2022). Experimental and numerical research on reinforced concrete slabs strengthened with POZD coated corrugated steel under contact explosive load. Int. J. Impact Eng..

[4] Schenker A., Anteby I., Gal E., Kivity Y., Nizri E., Sadot O., Michaelis R., Levintant O., Ben-Dor G. (2008). Full-scale field tests of concrete slabs subjected to blast loads. Int. J. Impact Eng..

[5] Dharmasena K.P., Wadley H.N., Xue Z., Hutchinson J.W. (2008). Mechanical response of metallic honeycomb sandwich panel structures to high-intensity dynamic loading. Int. J. Impact Eng..

[6] Stapczynski J.S. (1982). Blast injuries. Ann. Emerg. Med..

[7] Bartyczak S., Mock J. (2012). Versatile gas gun target assembly for studying blast wave mitigation in materials. AIP Conference Proceedings.

[8] Schleyer G.K., Lowak M.J., Polcyn M.A., Langdon G.S. (2007). Experimental investigation of blast wall panels under shock pressure loading. Int. J. Impact Eng..

[9] Thiagarajan G., Kadambi A.V., Robert S., Johnson C.F. (2015). Experimental and finite element analysis of doubly reinforced concrete slabs subjected to blast loads. Int. J. Impact Eng..

[10] Rodrígueznikl T. (2006). Experimental Simulations of Explosive Loading on Structural Components: Reinforced Concrete Columns with Advanced Composite Jackets. Ph.D. Thesis.

[11] Oesterle M.G. (2009). Blast Simulator Wall Tests: Experimental Methods and Mitigation Strategies for Reinforced Concrete and Concrete Masonry.

[12] Gram M.M., Clark A.J., Hegemier G.A., Seible F. (2006). Laboratory simulation of blast loading on building and bridge structures. Structures Under Shock and Impact Ix.

[13] Freidenberg A. (2013). Advancements in Blast Simulator Analysis Demonstrated on a Prototype Wall Structure. Ph.D. Thesis.

[14] Huson P. (2012). Experimental and Numerical Simulations of Explosive Loading on Structural Components: Composite Sandwich Connections. Ph.D. Thesis.

[15] Wolfson J.C. (2008). Blast Damage Mitigation of Steel Structures from Near-Contact Charges. Ph.D. Thesis.

[16] Paul S.C., Lv P., Xiao Y.-J., An P., Liu S.-Q., Luo H.-S. (2006). Thalidomide in rat liver cirrhosis: Blockade of tumor necrosis factor-alpha via inhibition of degradation of an inhibitor of nuclear factor-kappaB. Pathobiology.

[17] Peroni M., Solomos G., Caverzan A., Larcher M., Valsamos G. (2015). Assessment of dynamic mechanical behaviour of reinforced concrete beams using a blast simulator. EPJ Web Conf..

[18] Marco P., George S., Pierre P., Alessio C. (2015). Electrical Blast Simulator (e-BLAST): Design, Development and First Operational Tests.

[19] Hetherington J., Smith P. (2014). Blast and Ballistic Loading of Structures.

[20] Krauthammer T. (2008). Modern Protective Structures.

[21] Tekalur S.A., Bogdanovich A.E., Shukla A. (2009). Shock loading response of sandwich panels with 3-D woven E-glass composite skins and stitched foam core. Compos. Sci. Technol..

[22] Kinney G.F., Graham K.J. (1985). Explosive Shocks in Air.

[23] Eswaran M., Parulekar Y.M., Reddy G.R., Reddy G.R., Muruva H.P., Verma A.K. (2019). Introduction to Structural Dynamics and Vibration of Single-Degree-of-Freedom Systems. Textbook of Seismic Design: Structures, Piping Systems, and Components.

[24] U.S. Departments of the Army, the Navy and the Air Force (1990). Structures to Resist the Effects of Accidental Explosions (TM 5-1300/NAVFAC P-397/AFR 88-22, Revision 1).

[25] Chopra A. (2005). Dynamics of Structures.

[26] Zhou X.Q., Kuznetsov V.A., Hao H., Waschl J. (2008). Numerical prediction of concrete slab response to blast loading. Int. J. Impact Eng..

[27] Rasouli A., Toopchi-Nezhad H. (2020). The influence of confined water on blast response of reinforced concrete slabs: Experimental investigation. J. Build. Eng..

[28] Wu J., Zhou Y., Zhang R., Liu C., Zhang Z. (2020). Numerical simulation of reinforced concrete slab subjected to blast loading and the structural damage assessment. Eng. Fail. Anal..

[29] Wang W., Zhang D., Lu F., Tang F., Wang S. (2013). Pressure-impulse diagram with multiple failure modes of one-way reinforced concrete slab under blast loading using SDOF method. J. Cent. South Univ..

[30] Departments of the Army, Air Force, and Navy and the Defense Special Weapons Agency (1997). Design and Analysis of Hardened Structures to Conventional Weapons Effects, TM 5-855-1/AFPAM 32-1147(I)/NAVFAC P-1080/DAHSCWEMAN-97.

[31] Shuaib M., Daoud O. (2015). Numerical Modelling of Reinforced Concrete Slabs under Blast Loads of Close-in Detonations Using the Lagrangian Approach. J. Phys. Conf. Ser..

[32] Trentacoste M. (2007). Users Manual for LS-DYNA Concrete Material Model 159.

[33] (2015). LS-DYNA Keyword User’s Manual (LS-DYNA R8.0).

[34] Blatz P.J., Ko W.L. (1962). Application of Finite Elastic Theory to the Deformation of Rubbery Materials. J. Rheol..

[35] Murray Y.D. Theory and Evaluation of Concrete Material Model 159. Proceedings of the 8th International LS-DYNA Users Conference.

[36] Parfilko Y. (2017). Study of Damage Progression in CSCM Concretes Under Repeated Impacts. Master’s Thesis.

[37] ANSYS (2008). ANSYS LS-DYNA User’s Guide.

